# Guselkumab, Risankizumab, and Tildrakizumab demonstrate parallel effectiveness and safety in psoriasis treatment: a head-to-head comparative study in real clinical practice^☆^

**DOI:** 10.1016/j.abd.2024.04.004

**Published:** 2024-08-10

**Authors:** Miguel Mansilla-Polo, Antonio Sahuquillo-Torralba, Conrad Pujol-Marco, Guillermo Bargues-Navarro, Rafael Botella-Estrada

**Affiliations:** a Department of Dermatology, Hospital Universitario y Politécnico La Fe, Valencia, España; b Department of Dermatology, Instituto de Investigación Sanitaria La Fe (IIS La Fe), Valencia, España; c Department of Dermatology, Facultad de Medicina, Universidad de Valencia, Valencia, España

Dear Editor,

Traditionally, the treatment of psoriasis has relied on the use of topical treatments and classical drugs, such as methotrexate, acitretin, or cyclosporine, with varying results and a high burden of side effects. However, in recent years, the emergence of biological and small molecule therapies has revolutionized its treatment.^1^ Among the biological agents, we have Interleukin (IL) 23 inhibitors, which play a key role in the pathogenesis of psoriasis.^2^ Currently, we have three approved anti-IL-23 drugs: risankizumab, tildrakizumab, and guselkumab. The number of studies evaluating these drugs in real clinical practice for psoriasis is limited. Even scarcer are comparative studies among the three alternatives.

This article presents the results of a retrospective single-center study involving all patients with psoriasis treated with anti-IL-23 drugs. The objectives were to describe the response in terms of the effectiveness and safety of risankizumab, tildrakizumab, and guselkumab in real clinical practice. Additionally, we aimed to compare the response among the three drugs to detect differences in outcome measures among the alternatives.

Patients with psoriasis treated with anti-IL-23 drugs in a tertiary hospital between 2015 and 2020 were retrospectively collected. They were followed for one year. Outcome measures at 16, 24, and 48–52 weeks were recorded in terms of PASI (Psoriasis Area and Severity Index), BSA (Body Surface Area), IGA (Investigator’s Global Assessment), and DLQI (Dermatology Life Quality Index), as well as reported adverse events. Patients with less than 16 weeks of follow-up, those from a clinical trial, or those in whom at least 50% of the variables recorded in the study were not available were excluded.

Ninety one patientes were included: 42 patients received Risankizumab, 37 Guselkumab, and 12 Tildrakizumab. Table 1 depicts the baseline characteristics of the three groups, as well as some outcome measures not included in Fig. 1. Fig. 1 illustrates the response evolution in terms of PASI, BSA, IGA, and DLQI. The three groups were comparable regarding their baseline characteristics and initial clinical severity of the disease. Most patients were males, around 60 years old, and overweight. Approximately half and one-third of the patients were smokers, ex-smokers, drinkers or ex-drinkers, respectively. Eleven percent had psoriatic arthritis under Rheumatology follow-up, 10% had a history of neoplasia, and 7% had inflammatory bowel disease.

**Table 1:** Description of the sample and outcome variables not included in Fig. 1.

		Current drug (January 2024)			
		Guselkumab (n = 37)	Risankizumab (n = 42)	Tildrakizumab (n = 12)	p-value
**Continuous variables: median (first-third quartile)**	Age (years)	61 (47‒67)	55 (47‒64)	56.5 (49‒63.5)	0.752
	Weight (kg)	77 (66.5‒88.5)	85 (70‒95)	80.5 (71‒93.75)	0.283
	Size (cm)	171 (161‒178)	170 (162.3‒175)	165 (163.5‒179.5)	0.923
	Body mass index (kg/m^2^)	25.84 (23.99‒29.13)	29.33 (25.14‒33.70)	28.12 (23.91‒32.13)	0.116
	Number of biological drugs prior to current Interleukin (IL)-23 inhibitor drug	1 (1‒2)	1 (1‒2)	1 (0‒1)	0.245
	Number of years with psoriasis until current IL-23 inhibitor drug	12 (9‒20.5)	14.5 (8.5‒21.5)	12 (8.5‒16)	0.705

**Categorical variables: number of patients (% of the total of each drug)**	Sex	Men: 20 (54%)	Men: 23 (55%)	Men: 7 (58%)	0.967
		Women: 17 (46%)	Women: 19 (45%)	Women: 5 (42%)	
	Tobacco	Non-smoker: 16 (43%)	Non-smoker: 16 (41%)	Non-smoker: 4 (36%)	0.522
		Ex-smoker: 6 (16%)	Ex-smoker: 2 (5%)	Ex-smoker: 2 (18%)	
		Current smoker: 15 (41%)	Current smoker: 21 (54%)	Current smoker: 5 (45%)	
	Alcohol	Non-alcohol: 27 (73%)	Non-alcohol: 34 (81%)	Non-alcoholic: 8 (67%)	0.412
		Former alcohol consumer: 2 (5%)	Former alcohol consumer: 0 (0%)	Former alcohol consumer: 0 (0%)	
		Current alcohol drinker: 8 (22%)	Current alcohol drinker: 8 (19%)	Current alcohol drinker: 4 (33%)	
	Arterial hypertension	No: 24 (65%)	No: 28 (67%)	No: 11 (92%)	0.196
		Yes: 13 (35%)	Yes: 14 (33%)	Yes: 1 (8%)	
	Mellitus diabetes	No: 26 (70%)	No: 32 (76%)	No: 10 (83%)	0.596
		Yes: 11 (30%)	Yes: 10 (24%)	Yes: 2 (17%)	
	Dyslipidemia	No: 21 (57%)	No: 25 (60%)	No: 6 (50%)	0.840
		Yes: 16 (43%)	Yes: 17 (42%)	Yes: 6 (50%)	
	Hepatic steatosis	No: 12 (52%)	- No: 4 (17%)	No: 5 (56%)	0.032^a^
		Yes: 11 (48%)	Yes: 19 (83%)	Yes: 4 (44%)	
	Psoriasic arthritis	No: 29 (78%)	No: 39 (93%)	No: 12 (100%)	0.069
		Yes: 8 (22%)	Yes: 3 (7%)	Yes: 0 (0%)	
	inflammatory bowel disease	No: 36 (97%)	No: 36 (86%)	No: 12 (100%)	0.112
		Yes: 1 (3%)	Yes: 6 (14%)	Yes: 0 (0%)	
	History of neoplasia	No: 32 (86%)	No: 38 (90%)	No: 11 (92%)	0.895
		Yes: 5 (14%)	Yes: 4 (10%)	Yes: 1 (8%)	
	Scalp involvement (CC)	No: 17 (47%)	No: 22 (54%)	No: 6 (55%)	0.829
		Yes: 19 (53%)	Yes: 19 (46%)	Yes: 5 (45%)	
	Nail damage	No: 36 (100%)	No: 39 (95%)	No: 8 (89%)	0.331
		Yes: 0 (0%)	Yes: 2 (5%)	Yes: 1 (11%)	
	Genital involvement	No: 31 (86%)	No: 36 (88%)	No: 7 (78%)	0.816
		Yes: 5 (14%)	Yes: 5 (12%)	Yes: 2 (22%)	
	Affectation in folds	No: 23 (62%)	No: 28 (68%)	No: 5 (50%)	0.585
		Yes: 14 (38%)	Yes: 13 (32%)	Yes: 5 (50%)	
	Biologics prior to current IL-23 inhibitor	No: 8 (22%)	No: 8 (19%)	No: 4 (33%)	0.612
		Yes: 29 (78%):	Yes: 34 (81%):	Yes: 8 (67%):	
		• Etanercept: 3 (8%)	• Etanercept: 3 (9%)	• Etanercept: 2 (17%)	
		• Adalimumab: 17 (46%)	• Adalimumab: 12 (35%)	• Adalimumab: 6 (50%)	
		• Infliximab: 0	• Infliximab: 3 (9%)	• Infliximab: 0 (0%)	
		• Ustekinumab: 4 (24%)	• Ustekinumab: 5 (12%)	• Ustekinumab: 0 (0%)	
		• Secukinumab: 8 (22%)	• Secukinumab: 13 (31%)	• Secukinumab: 0 (0%)	
		• Ixekizumab: 3 (8%)	• Ixekizumab: 3 (9%)	• Ixekizumab: 0 (0%)	
		• Brodalumab: 2 (5%)	• Brodalumab: 7 (17%)	• Brodalumab: 0 (0%)	
				• Guselkumab: 0 (0%)	
				• Tildrakizumab: 0 (0%)	
				• Risankizumab: 0 (0%)	

**Result variables not included in the figures**^b^	CC response (in patients with CC involvement)	No: 0 (0%)	No: 2 (12%)	No: 1 (20%)	0.353
		Yes: 16 (100%)	Yes: 14 (88%)	Yes: 4 (80%)	
	Nail response (in patients with nail involvement)	Does not apply	No: 0 (0%)	No: 0 (0%)	Not calculable
			Yes: 2 (100%)	Yes: 1 (100%)	
	Genital response (in patients with genital involvement)	No: 1 (20%)	No: 1 (20%)	No: 0 (0%)	Not calculable
		Yes: 4 (80%)	Yes: 4 (80%)	Yes: 2 (100%)	
	Response in folds (in patients with involvement in folds)	No: 1 (8%)	No: 1 (10%)	No: 1 (20%)	0.984
		Yes: 12 (92%)	Yes: 9 (90%)	Yes: 4 (80%)	
	Dosage optimization^c^	No: 31 (86%)	No: 38 (93%)	No: 8 (67%)	0.058
		Yes: 5 (14%):	Yes: 3 (7%):	Yes: 4 (33%):	
		• With adequate response: 5 (100%)	• With adequate response: 2 (67%)	• With adequate response: 3 (75%)	
		• No appropriate response: 0 (0%)	• No appropriate response: 1 (33%)	• No appropriate response: 1 (25%)	

a After applying the Bonferroni test, no differences were found between the three drugs.

b Response was defined as disappearance of at least 75% of the previous lesions (BSA improvement ≥ 75%).

c Adequate response was defined as, at least, maintenance of PASI and BSA after therapeutic optimization (maintenance or stability). The optimization consisted of lengthening the therapeutic administration (1, 2, 3 or up to 4 weeks depending on the drug).

**Figure 1:**
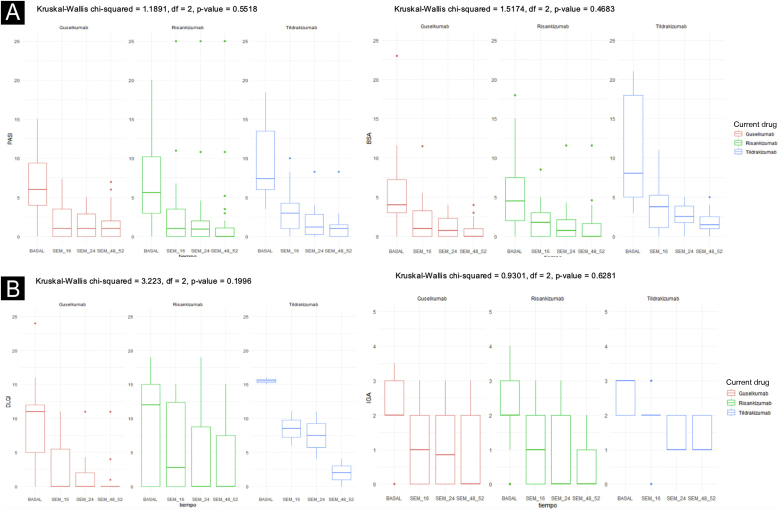
Results in terms of PASI (A), BSA (B), DLQI (C), and IGA (D). A clear trend of decreasing values for all 4 measures with the three drugs can be observed as the observation weeks progress.

Rapid and sustained improvements over time were observed in the studied variables with all three drugs (PASI, BSA, DLQI, IGA, nail response, scalp, genital, and fold involvement), with an excellent safety profile (an example of a patient treated with Risankizumab can be seen in Figure 2, Figure 3). To carry out the statistical study in Fig. 1, normality tests were conducted for all data using the Shapiro-Wilk test, and Non-Normality was detected. Therefore, to assess the 4 variables over time (Baseline Value ‒ week 48‒52 value) with respect to each of the three current drugs, a non-parametric test was employed: Kruskal-Wallis. Only two adverse effects (nausea and asthenia) were recorded in two different patients, both mild and not clearly related to treatment. In a head-to-head comparison, no statistically significant differences were found among the three drugs in any parameter, neither in terms of effectiveness nor safety.

**Figure 2:**
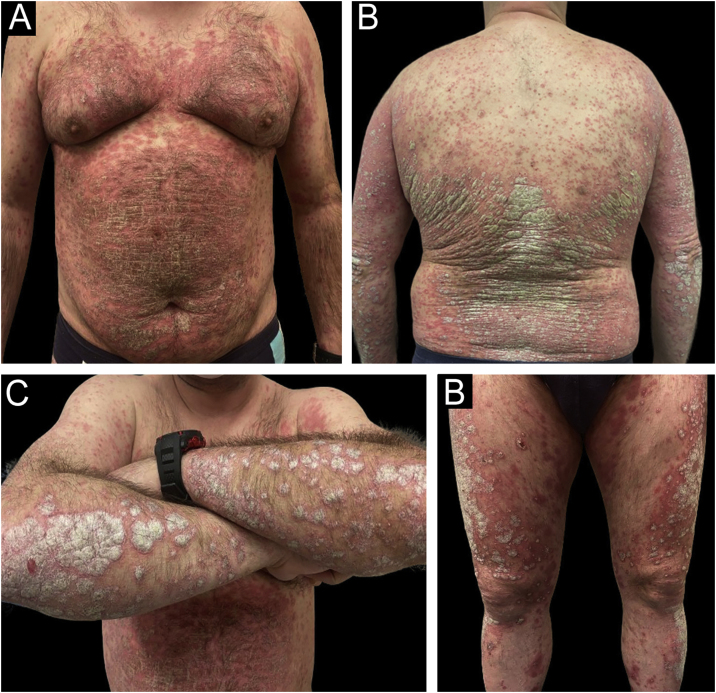
Example of a real patient prior to treatment with risankizumab. The involvement was generalized, with an initial staging of PASI 38.4; BSA 46%, and DLQI 28.

**Figure 3:**
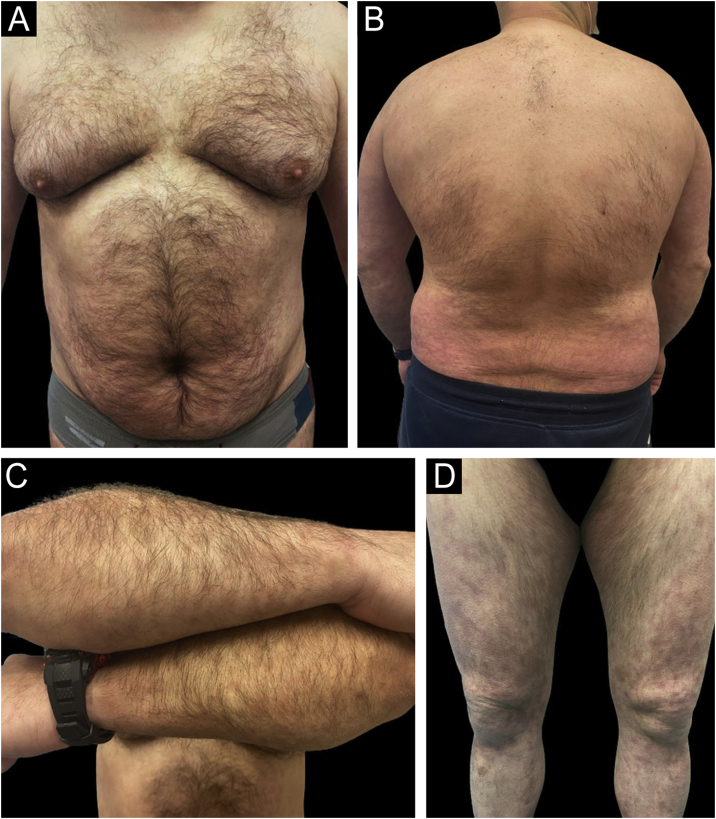
Example of a real patient (Patient of Fig. 2) after 6 months of treatment with risankizumab. The staging at 6 months was PASI 1.2; BSA 2%, and DLQI 4.

IL-23 plays a crucial role in the activation of T lymphocytes and the production of other proinflammatory cytokines, such as IL-17. By inhibiting IL-23, anti-IL-23 drugs reduce the underlying inflammatory response in psoriasis, thus decreasing the proliferation of keratinocytes and the formation of characteristic cutaneous plaques of the disease.^3^ The effectiveness and safety of the three approved anti-IL-23 drugs (risankizumab, tildrakizumab, guselkumab) have been demonstrated in clinical trials and real clinical practice.^4^, ^5^, ^6^ Our study’s results support this fact. Two previous studies, through indirect comparison analyses, suggest that the three alternatives would be comparable in terms of effectiveness and safety, although they suggest that Tildrakizumab might have slightly lower effectiveness.^7^, ^8^ A recent study, to our knowledge the only one to date comparing the different anti-IL-23 drugs in psoriasis, argues that all three present parallel effectiveness and safety, with no differences among them.^9^ Our results are similar, and we also did not find differences among the three groups, although the small sample size limits the statistical power of this comparison and the possibility of subgroup analysis (psoriasis severity, location, psoriatic arthritis, etc.).

The main limitations of our study are the small sample size, its retrospective nature, and its limited one-year treatment follow-up.

In conclusion, guselkumab, tildrakizumab, and risankizumab showed a reduction over time in biological measures (PASI, DLQI, BSA, IGA, scalp, nail, fold, genital response) in psoriasis treatment. No significant differences were found in the evolution of the effect over time among the different treatments: all three treatments worked equally. Studies with larger samples are needed to corroborate these results and delve into the presence of differences among the three IL-23 inhibitors currently used in psoriasis treatment.

## Financial support

None declared.

## Authors’ contributions

Miguel Mansilla-Polo: Managed clinical treatment and procedures, contributed to the development of this paper, had access to the data and played a role in writing this manuscript.

Guillermo Bargues-Navarro: Managed clinical treatment and procedures, contributed to the development of this paper, had access to the data and played a role in writing this manuscript.

Conrad Pujol-Marco: Managed clinical treatment and procedures, contributed to the development of this paper, had access to the data and played a role in writing this manuscript.

Rafael Botella-Estrada: Supervised the work, had access to the data and played a role in writing this manuscript.

Antonio Sahuquillo-Torralba: Supervised the work, had access to the data and played a role in writing this manuscript.

## Conflicts of interest

None declared.
